# A fatal case of viral sepsis and encephalitis in a child caused by human adenovirus type 7 infection

**DOI:** 10.1186/s12985-022-01886-z

**Published:** 2022-09-28

**Authors:** Hongwei Zhao, Yingchao Liu, Ziheng Feng, Qianyu Feng, Kechun Li, Hengmiao Gao, Suyun Qian, Lili Xu, Zhengde Xie

**Affiliations:** 1 Beijing Key Laboratory of Pediatric Respiratory Infection Diseases, Key Laboratory of Major Diseases in Children, Ministry of Education, National Clinical Research Center for Respiratory Diseases, National Key Discipline of Pediatrics (Capital Medical University), National Center for Children’s Health, Beijing Pediatric Research Institute, Beijing Children’s Hospital, Capital Medical University, Beijing, China; 2 Department of Paediatric Critical Care Medicine, National Center for Children’s Health, Beijing Children’s Hospital, Capital Medical University, Beijing, China; 3 Research Unit of Critical Infection in Children, Chinese Academy of Medical Sciences, 2019RU016, Beijing, China

**Keywords:** Human adenovirus 7, Encephalitis, Virus isolation

## Abstract

Adenoviruses are highly prevalent pathogens responsible for a wide range of clinical diseases, including respiratory tract infection, acute gastroenteritis, and conjunctivitis. However, adenovirus infection is rarely associated with central nervous system involvement. Here, we report a fatal viral sepsis and encephalitis in a child caused by a human adenovirus type 7 infection. We detected human adenovirus type 7 in the patient’s nasopharyngeal swab, blood, and cerebrospinal fluid. Our findings indicate clinicians should be aware of the possible central nervous system involvement in adenovirus infection.

Human adenoviruses (HAdVs) are nonenveloped, double-stranded DNA viruses consisting of more than 100 genotypes, which can be further subdivided into one of 7 (A–G) species [1]. HAdVs mainly cause respiratory, gastrointestinal, urinary, and ocular infections in adults and children. It has been demonstrated that HAdV species B is associated with worse clinical presentation and prognosis, and HAdV type 7 is commonly associated with severe lower respiratory tract diseases in particular [2]. Extremely sporadic previous studies have reported the presence of adenovirus in cerebrospinal fluid (CSF) and its causative relationships with encephalitis [3–5]. Adenovirus-related central nervous system (CNS) diseases cover a wide range, including meningitis, encephalitis, and encephalopathy. The prognosis of adenovirus-related CNS involvement is usually good [6], and risk factors associated with adverse clinical outcomes include seizures, coagulopathy, and young age [7]. No fatal cases of HAdV type 7-related encephalitis have been reported in this decade. Here, we present a fatal case of viral sepsis and encephalitis caused by HAdV type 7 infection, where we detected HAdV type 7 in the patient’s nasopharyngeal swab, blood, and CSF.

Six days before admission, a 20-month-old boy experienced a nonproductive cough and fever (maximum temperature, 40.5 °C) with no previous medical history. Chest computed tomography (CT) showed patchy consolidation in the bilateral lung lobes. He was prescribed ceftriaxone and ibuprofen, but the medication did not relieve his symptoms. He was then transferred to the emergency department of our hospital. On admission, his temperature was 38.5 °C, blood pressure was 78/63 mmHg, heart rate was 132 beats per minute, respiratory rate was 32 breaths per minute, and oxygen saturation was 93% while the patient was breathing ambient air. On physical examination, he showed tachypnoea and increased inspiratory effort with the chest wall, suprasternal, and subcostal retractions. Other physical findings were unremarkable. His white blood cell count was 7.32 × 10^9^/L (reference range, 4.1–11.3 × 10^9^/L), with 56.1% neutrophils (reference range, 31–70%) and 41.4% lymphocytes (reference range, 23–59%); his C-reactive protein level was 16 mg/L (reference range, < 8 mg/L); and his procalcitonin level was 0.93 ng/ml (reference range, < 0.25 ng/ml).

He was empirically treated with cefamandole and azithromycin intravenously to control the infection to improve airway condition with budesonide and ipratropium bromide nebulization. Five days after admission, he experienced an episode of convulsion with loss of consciousness, which lasted 1 min and ceased after intravenous use of 2 mg diazepam. He was intubated and on mechanical ventilation for worsening oxygen saturation (75% for SaO_2_, 55 breaths per minute) with cyanosis after convulsion. One hour after his first convulsion, the second episode began and could not be controlled by diazepam (3 mg). He was thus given a series of anti-epilepsy therapies, including 0.1 g of phenobarbital via intramuscular injection, 2 mg of midazolam via intravenous injection, and 5 ml of chloral hydrate via the rectum. The second episode lasted 12 min, and his Glasgow Coma Scale (GCS) score was 5 T (E1VTM4). He was then transferred to the pediatric intensive care unit (PICU) for supportive care. His CSF analysis revealed that his protein (548 mg/L, reference range, 20–450 mg/L) and glucose (5.2 mmol/L, reference range, 2.8–4.5 mmol/L) levels were increased. The chest CT image (Fig. 1A) showed no resolution of lung inflammation after five days of treatment, and there were no noteworthy findings in the cerebral CT image (Fig. 1B). The patient experienced recurrent and persistent convulsions requiring continuous intravenous midazolam and phenobarbital to control the seizures. His respiratory and CNS conditions continued to deteriorate. The patient was diagnosed with acute respiratory distress syndrome (ARDS) 8 days after admission. Nine days after admission, the patient’s heart rate dropped, and he developed ventricular fibrillation. The CRP and PCT levels significantly increased; thus, in combination with the confirmed pathogen, he was diagnosed with viral sepsis. His guardian refused ECMO therapy, and the patient died of ARDS 10 days after admission.

**Fig. 1:**
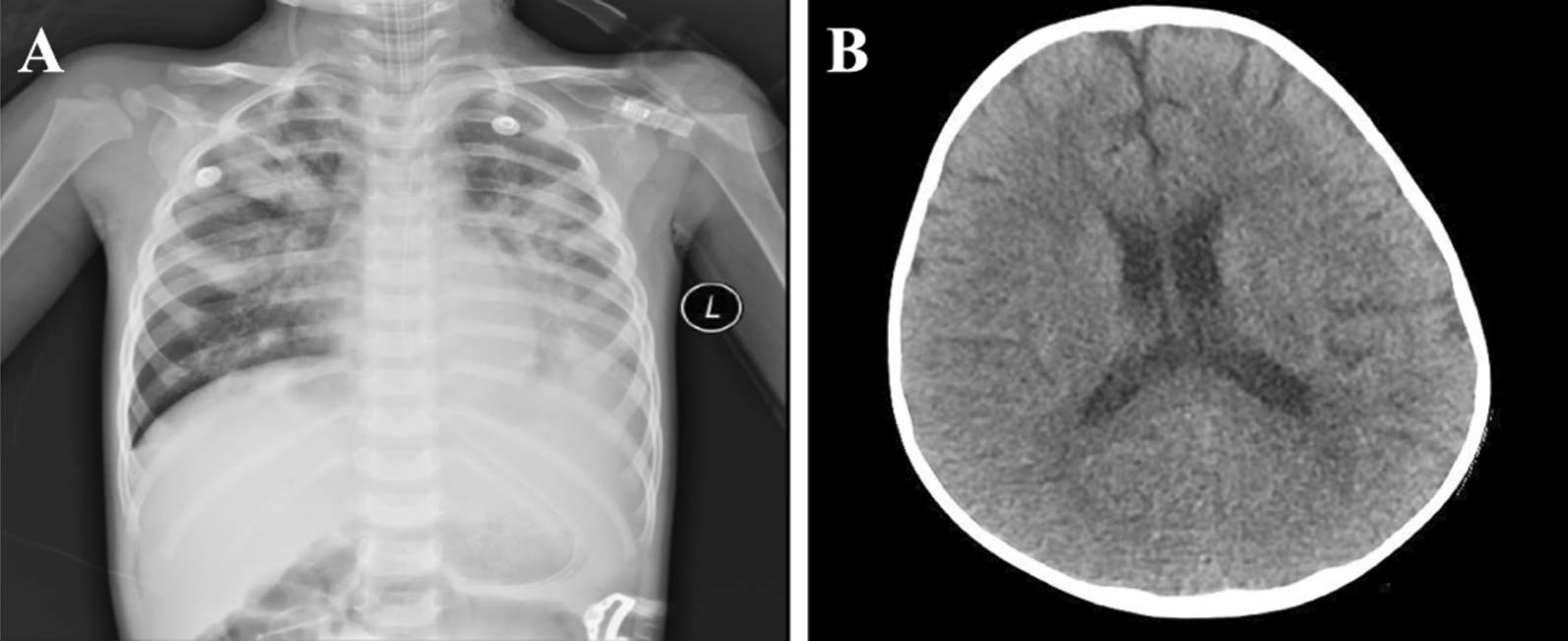
**A** Chest computed tomography image obtained 5 days after admission. **B** Cerebral computed tomography image obtained 5 days after admission

We detected adenovirus in his nasopharyngeal swab and blood sample (collected on day 2 after admission) by real-time PCR and in his CSF (collected on day 5 after admission) by mNGS, which revealed 53 HAdV type 7 sequences with 43.59% coverage. Real-time PCR-positive nasopharyngeal swab samples were inoculated onto A549 cells for virus isolation. We infected SK-N-SH cells (human neuroblastoma cells) with the isolated virus, and cytopathic effects could be seen 48 h post-inoculation (Fig. 2). Complete genome sequences of 35,232 bp were identified using a Sanger primer-walking method. The whole-genome sequence has been deposited in GenBank under accession number OP270254. Phylogenetic analysis was performed on the complete sequence of our strain and compared with other available sequences of HAdV type 7 in GenBank, indicating that our strain did not form a distinct clade (Fig. 3). Phylogenetic analysis of the genes encoding the three most important surface proteins (hexon, fiber, and penton base) also yielded results similar to the complete sequence. Next, we performed genome sequence analysis on the hexon, fiber, and penton base genes, identifying 3 specific amino acid missense mutations in penton base genes (H477P, H480P, and S487F, Fig. 4A) compared to the reference sequence (accession number AC_000018.1). All three mutations have not been reported previously. We performed three-dimensional modeling of protein homology structures based on the amino acid sequence of the reference strain and our isolated strain (Fig. 4B, C, respectively) following the SWISS-MODEL (https://swissmodel.expasy.org) protocol [8]. The homology models suggested that the 3 specific amino acid mutations found in our strain had no differences compared with the reference strain in terms of protein structure and function. However, further studies are needed to verify the results.

**Fig. 2:**
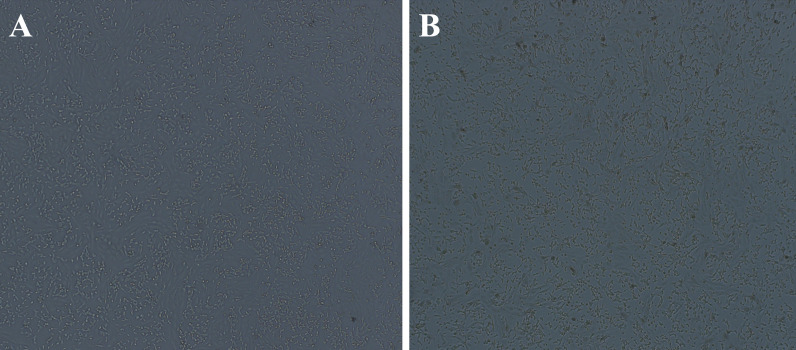
Cytopathic effect (CPE) in SK-N-SH cells 48 h post-inoculation. **A** Control inoculated with DMEM. **B** Inoculated with isolated HAdV 7

**Fig. 3:**
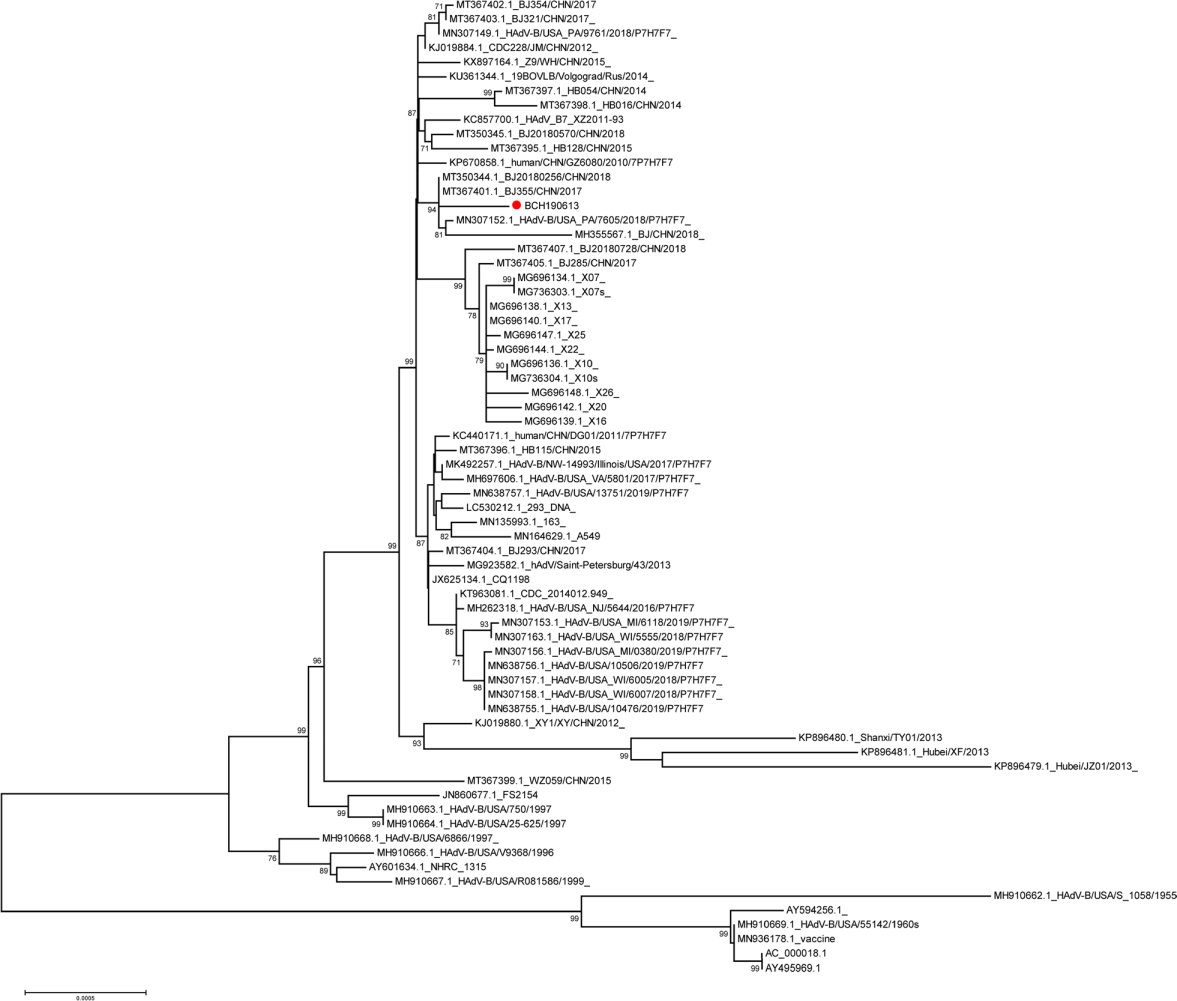
Phylogenetic analysis of HAdV 7. The strain identified in our study (red circle) and other available sequences of HAdV 7 in GenBank were analyzed. A phylogenetic tree was constructed using the neighbor-joining method (Kimura’s two-parameter) with 1000 bootstrap values

**Fig. 4:**
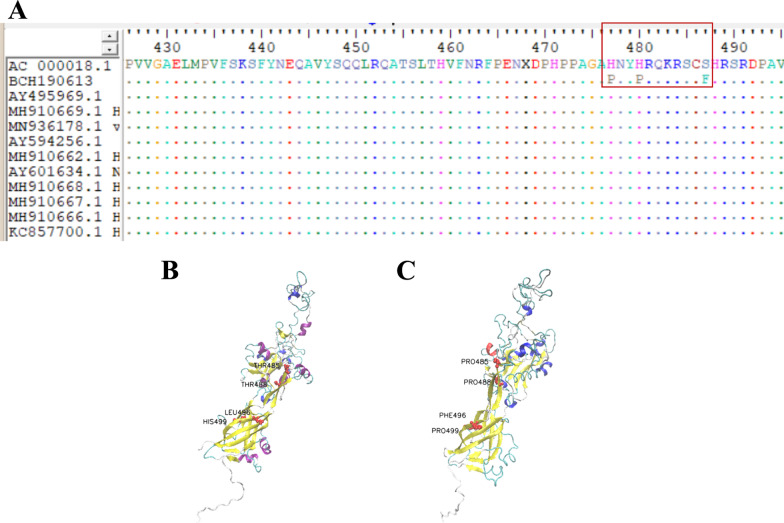
**A** Genome sequence analysis of the penton base genes revealed 3 specific amino acid missense mutations. The penton base homology model of the reference strain (**B**) and our isolated strain (**C**)

Adenoviruses rarely insult the CNS since their receptors, such as desmoglein 2 and CD46, are expressed at low levels in brain tissue [9]. Based on the small number of reported cases, it is difficult to determine which serotypes are likely to cause CNS infection. However, a study indicated that serotype 7 accounted for 56% of all adenovirus-related CNS diseases, with death or neurological sequalae occurring in 40% of HAdV type 7 encephalitis/encephalopathy [7]. Thus, we cannot determine whether the propensity of HAdV type 7 for CNS involvement or the specific mutations that might enable its CNS tropism and promote virus virulence led to the CNS infection and fatal outcome in our case.

Host factors play an important role in virus pathogenesis. Blood–brain barrier (BBB) integrity is crucial to prevent viral CNS infection. Whole-exome sequence analysis revealed that a potential pathogenic mutation (KDR) might have an important role in the pathogenesis of our patient. KDR encodes vascular endothelial growth factor receptor 2 (VEGFR-2), whose downstream signaling leads to endothelial cell proliferation, survival, and migration via different pathways [10]. KDR mutations might affect blood vessel formation throughout the body and subsequently compromise BBB integrity, allowing the virus to cross the BBB into the CNS [11]. In the physiological state, the BBB is highly selectively semipermeable, constricting viruses from invading the CNS. However, inflammation can lead to BBB disruption, which allows the virus to insult the CNS. Widespread BBB disruption could be an etiology in epileptogenesis and could facilitate enduring neuronal hyperexcitability [12]. Subsequent CNS inflammatory responses and seizures could promote BBB opening and breakdown from the inside [13]. Thus, as seen in our patient, BBB disruption, inflammation, and seizures interact as both cause and effect and work as positive feedback in the pathogenesis of recurrent seizures.

There are several limitations to our study. Although we detected HAdV type 7 in the patient’s CSF using the mNGS method, we did not have enough CSF samples for virus isolation. Meanwhile, we only performed one cerebral CT scan at the onset of convulsions, which could not provide further information on the development of intracranial pathology. In conclusion, our findings indicate that HAdV type 7 can be an underlying etiology of CNS infection and suggest that clinicians should always be aware of possible CNS insults in HAdV-infected patients.

## Data Availability

The datasets supporting the conclusions of this article are included within the article.
